# Terrestrial Compound Protein Replacing Dietary Fishmeal Improved Digestive Enzyme Activity, Immune Response, Intestinal Microflora Composition, and Protein Metabolism of Golden Pompano (*Trachinotus ovatus*)

**DOI:** 10.1155/2023/2716724

**Published:** 2023-10-04

**Authors:** Yongcai Ma, Zeliang Su, Fang Chen, Chao Xu, Kunsheng Jiang, Wenqiang An, Guanrong Zhang, Dizhi Xie, Shuqi Wang, Yewei Dong, Yuanyou Li

**Affiliations:** ^1^College of Marine Sciences of South China Agricultural University and Guangdong Laboratory for Lingnan Modern Agriculture, Guangzhou 510642, China; ^2^Guangdong Provincial Key Laboratory of Marine Biotechnology, Shantou University, Shantou 515063, China; ^3^College of Animal Science and Technology of Zhongkai University of Agriculture and Engineering, Guangzhou 510225, China

## Abstract

Terrestrial compound protein (Cpro) can be potentially used to replace fishmeal (FM) in the marine carnivorous teleost, golden pompano (*Trachinotus ovatus*). Four isonitrogenous (45%) and isolipidic (12%) diets named FM30, AP80, PP80, and CP80 were formulated. FM30 (control) contained 30% FM and 25% basic protein, while AP80, PP80, and CP80 only contained 6% FM, where 80% FM and 25% basic protein of control diet were completely replaced by animal protein, plant protein, and Cpro, respectively. After golden pompano juveniles (initial weight: 10.32 ± 0.09 g) were, respectively, fed the four diets in floating sea cages for 10 weeks, the growth performance, intestinal digestive enzyme activity, and immune responses, protein metabolism indices of the CP80 group were similar to or better than those of the FM30 group (*P* > 0.05), and significantly better than those of the AP80 and PP80 groups. Specifically, the weight gain (WG), feed conversion ratio (FCR), activity of alanine transaminase (ALT), growth hormone (GH), and insulin-like growth factor-1 (IGF-1) contents of serum, mRNA level of interleukin-10 (*il*-10), zonula occludens-2 (*zo*-2), *claudin*-3, *claudin*-12, and eukaryotic translation initiation factor 4G (*eif*4*g*) were significantly higher, and the activity of *α*-amylase (AMS), lipase (LPS) in the foregut and midgut, interleukin-8 (*il*-8) expression in the intestine was significantly lower than that in the CP80 group, compared with those in AP80 and PP80 groups (*P* < 0.05). Moreover, the intestinal microflora composition of golden pompano fed with the CP80 diet was improved. Specifically, at the phylum level, the relative abundance of harmful bacterial strains cyanobacteria and TM7 of CP80 group was similar to those of FM30 group (*P* > 0.05), but was significantly lower than those of AP80 and PP80 groups (*P* < 0.05). At the genus level, the beneficial bacterial strains *Agrobacterium* and *Blantia* of CP80 group were also similar to those of FM30 group (*P* < 0.05), which were significantly higher than those of AP80 and PP80 groups, but the beneficial bacterial strains *Bifidobacterium* and *Devosia* of CP80 group were significantly higher than that in the other groups (*P* < 0.05). Besides, in diet CP80, the contents of amino acids and anti-nutritional factor, as well as the in vitro digestion rate were comparable to those of FM30, and the anti-nutritional factor content was between AP80 and PP80; total essential amino acids (EAAs) and methionine contents were higher than those in AP80, the glycine content was higher than that in PP80. Taken together, these results indicated that the CP80 diet had better amino acid composition and relatively low content of anti-nutritional factors, as well as high-digestion rate, and thus leads to the fish fed CP80 displaying improved effects in digestive enzyme activity, immune response, protein metabolism, and intestinal microbiota composition, which may be the important reasons to explain why that 80% of FM can be replaced by Cpro in the diet of golden pompano.

## 1. Introduction

With the rapid development of aquaculture, aquatic animal nutrition and feed has also become the focus of being paid attention [1]. Fishmeal (FM), is still the main protein source in commercial feeds for most carnivorous fish due to its advantages of amino acid balance, good adaptability, high digestibility, and low-anti-nutritional factor content [2, 3]. At present, the global marine fisheries produce about 80 million metric tons per year, in which although one third of the production is processed into FM and fish oil (FO) for the feed industries [2], it still fails to meet the demand of aquatic feed enterprises though, leading to the annual increase in FM prices [4]. In addition, widespread and rapid changes in climate and environmental systems due to human activities have resulted in FM shortages in marine resources and a significant increase in FM prices, posing major challenges to the aquaculture [5, 6]. Therefore, finding suitable substitute of dietary FM is very important for the sustainable development of aquaculture.

Up till now, lots of studies have been done on FM substitution, mainly using plant protein (such as soybean meal, soybean protein concentrate, and peanut meal) and animal protein (such as meat and bone meal, blood powder, and insect powders) as alternative sources of FM [7, 8]. However, replacing FM with a high proportion of animal protein sources usually resulting to an imbalance of amino acids, or a lack of certain essential amino acids (EAAs) in the feed [9, 10]. FM replaced with a high proportion of plant protein sources will increase the anti-nutritional factors (such as *β*-conglycinin, soybean antigen, soy glycinin, phytagglutinins, and plant trypsin inhibitor) in the feed [11], resulting in a decrease in the digestibility of aquatic animals to feed [12]. In addition, long-term feeding of low-FM feed can also induce changes in the composition of the intestinal flora of aquatic animals [13, 14], and negatively affect feed intake, digestive enzyme activity, intestinal microbial composition and health, immunity and protein metabolism, ultimately reducing their growth performance of aquatic animals [15–17]. Growth is the essential result of muscle cell proliferation and protein synthesis [18, 19], and low-FM intake in aquatic animals can adversely affect fish protein metabolism, which is mainly regulated by targets of the rapamycin (TOR) signaling pathway [20, 21]. At present, it is usually necessary to add 20%–30% FM to the formula feed of marine carnivorous fish, which seriously restricts its large-scale development [22]. Therefore, how to maintain the balance between FM replacement protein source and fish health, and improve the FM replacement level and increase the growth performance of fish has become the major problem in FM replacement applications.

Golden pompano (*Trachinotus ovatus*), a marine carnivorous fish which can be fed with artificial compound feed throughout the process, is cultivated in large quantities in southern China, and its output in 2021 has reached 243,908 tons [23, 24]. In previous years, at least 30% FM is needed in the commercial feed of golden pompano, which seriously restricts its large-scale farming [23, 25]. In recent years, lots of studies have been conducted on FM replacement in this fish using fermented soybean meal [26], soybean protein concentrate [27], cottonseed protein concentrate [28], poultry byproduct meal [29], corn gluten meal [30], and other single protein sources, which lead to the addition of dietary FM to be reduced to 16%–28%. Recently, our team developed a terrestrial compound protein (Cpro), which can replace 80% of dietary FM without reducing the growth performance of golden pompano [25, 31]. However, the reason and mechanism for the high proportion of Cpro in replacing FM are unclear. The essence of dietary FM substitution is to establish the nutritional balance of feed formula in the context of low FM [32], understanding the nutrient metabolism and growth mode of fish in the context of complex nutrients is the key to establishing a low-FM formula [33]. The factors limiting the substitution of FM in aquatic formula feed mainly include poor composition of amino acids, presence of anti-nutritional factors, and deficiency of growth-promoting factors, which lead to the low-digestible protein of feed for the aquatic animals [34]. Based on this, we speculate that the successful replacement of 80% dietary FM by Cpro without negatively affecting the growth performance of golden pompano may be related to the low content of anti-nutritional factors and balanced amino acid composition in the diet. To this end, four diets were designed to feed the golden pompano juveniles, which included a control diet (FM30) containing 30% FM and 25% basic protein, experimental diet AP80, PP80, and CP80 in which 80% FM and 25% basic protein of FM30 was replaced by animal protein, plant protein, and Cpro, respectively, so as to explore the reasons for the high proportion of Cpro replacing FM in feed by comparing the growth performance, digestive enzyme activity, intestinal microflora, immune response, and protein metabolism. The results may provide a theoretical basis for the application of Cpro and the preparation of low-FM diet for golden pompano, and also provide reference for the FM replacement of other marine teleosts.

## 2. Materials and Methods

### 2.1. Ethical Statement

The experimental procedure was approved by the Ethics of the Institutional Animal Care and Use Committee (IACUC) of Laboratory Animals of South China Agricultural University (Approval number: SYXK-2019-0136).

### 2.2. Experimental Diets

Four isoproteic (45%) and isolipidic (12%) experimental diets were formulated. Diet FM30 contained 30% FM (control), where 80% FM and 25% basic protein in diet FM30 were completely replaced by animal protein (AP80), plant protein (PP80), and Cpro (CP80), respectively. The Cpro of this study is the Cpro III of our team's previous research [25], which has been granted a South African patent. The specific composition and proportion of Cpro were not shown due to the protection of patent granted by Republic of South Africa (patent No. 2023/00178), which had a content of 54.11% crude protein and 6.34% crude lipid, respectively. To maintain the balance of EAA profile, L-lysine was added in the experimental diets. The method of diet preparation and storage can be seen in our study [35]. The experimental diets of formulations, proximate compositions are presented in Table 1; amino acids composition, anti-nutritional factor content, and in vitro digestibility are presented in Tables 2–5, respectively.

### 2.3. Analysis of Amino Acid Compositions, Anti-Nutritional Factor (ANFs), and In Vitro Digestion Rate of the Diets

The amino acid composition of diets was determined using the L-8900 amino acid analyzer (Hitachi, Tokyo, Japan) according to the method described by Ma et al. [25]. In brief, 30 mg of dried diet samples were hydrolyzed by 6 mol/L of HCL at 110°C in nitrogen atmosphere for 22–24 hr. The samples were then transferred and diluted into a 50 mL volumetric flask using 0.02 mol/L of HCl. After mixing, 1 mL of this solution was transferred to a glass dish and evaporated to remove any acid in a 65°C water bath. The acid-free samples were further dissolved with 2 mL of HCl (0.02 mol/L) and filtered through the 0.22 *μ*m membrane to remove impurities and any residue, and 1 mL of the solution was used for the determination of amino acids. The individual amino acids were determined by comparing their chromatographic peak heights with standards (013–08391, Wako, Tokyo, Japan). The amino acid results were shown as g/100 g of dry matter.

Dietary anti-nutritional factor (*β*-conglycinin, soybean antigen, soy glycinin, phytagglutinins, and plant trypsin inhibitors) contents of were quantified by the commercial ELISA kits (Shanghai Enzyme-linked Biotechnology Co., Ltd., China) following the manufacturer's protocols. The contents of the *β*-conglycinin, soybean antigen, and soy glycinin were presented as microgram per gram diet, and the contents of the phytagglutinins and plant trypsin inhibitors were presented as nanogram per gram diet.

The in vitro digestion rate of the diets was performed according to the method described by Liu [36] and Luten et al. [37] with slight modifications, as follows: the gastric or intestines of the juvenile golden pompano was fully ground in 4°C precooled mortar at a volume to weight ratio (*V*/*W*) of 1 : 1 and then added phosphoric acid buffer to achieve a volume to weight ratio (*V*/*W*) of 10 : 1, thoroughly, mix and centrifuge with a high-speed refrigerated centrifuge for 20 min (3,500 rpm, 4°C), and aspirate the supernatant as a digestive juices, which is used immediately. Gastric juice was extracted with a buffer solution of pH 2.5 and duodenal juice with a buffer solution of pH 8.0. In brief, 1.00 g of experimental diet and 15 mL of phosphoric acid buffer (0.2 M, pH 2.5) are mixed in an Erlenmeyer flask, 5 mL of gastric juice was added, 150 IU/mL of penicillin and thiostreptomycin 2 mL (one time every 2 hr), and the reaction was performed on an orbital shaker (50 rpm) for 6 hr. Subsequently, adjust the pH to 8.0 with 2 M NaOH, add 10 mL of phosphoric acid buffer (0.2 M, pH 8.0) and 10 mL of duodenal juice and continue incubating the mixture at 30°C on an orbital shaker (60 rpm) for 18 hr. Finally, 6 mL of 50% trichloroacetic acid was added to stop the enzymatic hydrolysis reaction, let stand for 30 min, filter with a quantitative filter paper of constant, weight and rinse the residue with 10 mL of ethanol and acetone, respectively. Bake in an oven at 105°C for 4 hr, determine the digestibility in vitro of the diet.

### 2.4. Feeding Trail

*T. ovatus* juveniles (the average weight and body length of each fish are about 2.50 g and 2.5–3.0 cm, respectively) obtained from a local fish hatchery (Nanao, Guangdong Province, China) were acclimated to the experimental conditions for 2 months by feeding a commercial diet (46% protein, 13% lipid, Haiyi feed company, Zhuhai, Guangdong, China). Then, 420 fish were randomly distributed into 12 floating cages (1.0 × 1.0 × 1.5 m) at the density of 35 fish per cage and acclimatized to the experimental conditions for 1 week and fed twice daily with a mixture of the four experimental diets in equal amounts. Finally, 30 fish of similar size were selected and weighed (average initial weight: 10.32 ± 0.09 g) in each floating cage for the formal experiment. The fish were fed to apparent satiation twice a day (6:00 and 17:00) for 10 weeks. During the feeding trial, the temperature of the seawater ranged from 27 to 30°C, the salinity fluctuated between 30–33g/L, and the dissolved oxygen was 5.0–8.0 mg/L.

### 2.5. Sampling Procedures

At the end of the feeding trial, the fish were fasted for 24 hr and anesthetized with 0.01% 2-phenoxyethanol (Sigma–Aldrich). All fish in each cage were counted and weighed. Then, two fish from each cage were measured body length. Blood was collected from the caudal vein into 1.5 mL centrifuge tubes with heparin-pretreated injection syringes and stored at 4°C for 3 hr, then immediately centrifuged at 3,000x *g* for 10 min at 4°C to collect serum for analysis. The weights of whole fish, viscera and liver were recorded for the determination of hepatic somatic index (HSI) and visceral somatic index (VSI). The liver and intestinal tissues were frozen in liquid nitrogen and stored at −80°C for biochemical analysis. Additionally, the gut content of two fish per cage was collected from the foregut region to the hindgut region according to the methods of Xu et al. [38], and then transferred to 1.5 mL sterile tubes for the analysis of the intestinal microbiota.

### 2.6. Analysis of Hormonal Contents, Digestive Enzyme, and Protein Metabolism Enzyme Activity

Fish growth hormone (GH, product code: SP28699) and insulin-like growth factor-1 (IGF-1, product code: SP28487) were determined in the serum of fish using their corresponding ELISA kits from Wuhan Saipei Biotechnology Co., Ltd. The activities of intestinal *α*-amylase (product code: C016-1-1), lipase (LPS, product code: A054-2-1), chymotrypsin (CHT, product code: A080-3-1), and pepsin (product code: A080-1), as well as serum and liver aspartate aminotransferase (AST, product code: C0010-2-1), alanine transaminase (ALT, product code: C009-2-1), xanthine oxidase (XOD, product code: A022-1-1) and succinic dehydrogenase (SDH, product code: A022-1-1), and the contents of total protein (TP, product code: A045-4), total amino acids (T-AA, product code: A026-1-1) were determined by the commercial kits (Nanjing Jiancheng Bioengineering Co., China).

### 2.7. Intestinal Microbiota Community Discovery and Analysis

The intestinal community of the 16S rRNA sequencing was performed following the methods of Xu et al. [38]. In brief, the V3–V4 region of the bacterial 16S rRNA gene was amplified by PCR (95°C for 2 min and followed by 30 cycles of 10s denaturation at 98°C, a 30s annealing step at 62°C, and a 30s extension step at 68°C, and then a final extension of 5 min at 72°C) using the 515f/806 r primer. 515f: GTGCCAGCMGCCGCGGTAA; 806r: GGACTACHVGGGTWTCTAAT. High-throughput sequencing was performed by an Illumina MiSeq platform according to the manufacturer's protocols provided using Suzhou PANOMIX Biomedical Tech. Co., Ltd., (China).

The data were filtered and the chimera was removed, and then bioinformatics analysis was performed using USEARCH software, with operational taxonomic units (OTUs) with 97% similarity to identify and remove chimeric sequences [39]. The most abundant labeled sequence was selected as the representative sequence within each cluster. Subsequently, the representative sequence for each OTU was screened and the differences of the dominant species in the groups were determined [40]. The Alpha diversity index Chao, Coverage, Shannon and Simpson were calculated using QIIME (v1.7.0) and displayed using R software (v2.15.3). The Chao index was used to identify community richness, the Shannon and Simpson indices to identify community diversity, and the Coverage index was used to evaluate the depth of the characterized sequencing [41].

### 2.8. Real-Time Quantitative PCR Analysis

Total RNA was extracted from the frozen intestine and live tissue samples using Trizol Reagent (Invitrogen) according to the manufacturer's instructions. The expression levels of target genes were determined by quantitative real-time PCR with SYBR Green (Bio-Rad, USA). The reaction system included 1 *μ*L cDNA, 0.4 *μ*L forward and reserve primers (10 mmol/*μ*L), 5.0 *μ*L SYBR, and 3.2 *μ*L double distilled water. The primers of the genes were presented in Table 5. Gene expression levels were quantified relative to the expression of *β*-actin using the 2^−*ΔΔ*Ct^ methods [42, 43].

### 2.9. Data Statistical Analysis

The data were expressed as means ± SEM. All data were analyzed using one-way analysis of variance (ANOVA) by SPSS 22.0 software. *P* < 0.05 was considered to be significant.

## 3. Results

### 3.1. Amino Acid Profile, Anti-Nutritional Factors, and In Vitro Digestion Rate of Diets

The amino acid profile of the diets was presented in Table 2. The EAAs contents of CP80 diets were similar to those of FM30 diets, and the contents of total EAAs, isoleucine, leucine, and phenylalanine of CP80 were higher than those in AP80 diets, while methionine contents were higher than that in the PP80 diet, as for non-EAAs, the content of glutamic acid was higher than that in AP80 diet, and the content of glycine was higher than that in PP80 diets. The contents of *β*-conglycinin, soybean antigen, soy glycinin, phytagglutinins, and plant trypsin inhibitor in the CP80 diets were between the diets PP80 and FM30 (Table 3). These contents of anti-nutritional factors of the PP80 diets were higher than those of the other groups. The lowest values of *β*-conglycinin, soybean antigen, soy glycinin, and phytagglutinins were observed in the AP80 diets. The in vitro digestion rate of dry matter, crude protein, and crude lipid of diet CP80 was similar to that of diet FM30, and higher than those of diets AP80 and PP80 (Table 4).

### 3.2. Growth Performance and Feed Utilization

The parameters of growth performance and feed utilization were shown in Table 6. There were no significant differences in SR, HSI, and CF among all the groups (*P* > 0.05). However, the final mean body weight (FBW) and weight gain (WG) of the CP80 group were not significantly different from those of the FM30 group (*P* > 0.05), but were significantly higher than those of the AP80 and PP80 groups (*P* < 0.05). In addition, the feed conversion ratio (FCR) of the CP80 group showed no significant difference to those of the FM30 and AP80 groups (*P* > 0.05), and it was significantly lower than that of the PP80 group. The VSI of the CP80 group showed no significant difference to than that of the other groups (*P* > 0.05).

### 3.3. Intestinal Enzymes Activities

The digestive enzyme activities of the foregut, midgut, and hindgut were shown in Table 7. In the foregut, the activities of CHT, LPS, and *α*-amylase (AMS) of the CP80 group showed no significant difference from those of the FM30 group (*P* > 0.05), while PEP activity was significantly higher than that of the FM30 and PP80 groups, AMS and CHT activity was significantly lower than that of the AP80 and PP80 groups (*P* < 0.05). In the midgut, PEP and CHT activity of the CP80 group showed no significant difference from that of the FM30 group (*P* > 0.05), and were significantly lower than that of the PP80 group, LPS was significantly higher than that of the FM30 group, AMS was significantly higher than that of the FM30 and AP80 groups (*P* < 0.05). In hindgut, the activities of PEP and LPS of the CP80 group were significantly lower than those of the FM30 group, PEP activity was significantly lower than that of the PP80 group, CHT activity was significantly higher than that of the AP80 group, and AMS activity was significantly higher than that of the FM30 group (*P* < 0.05).

### 3.4. Analyses of Intestinal Microbiota

**Figure 1:**
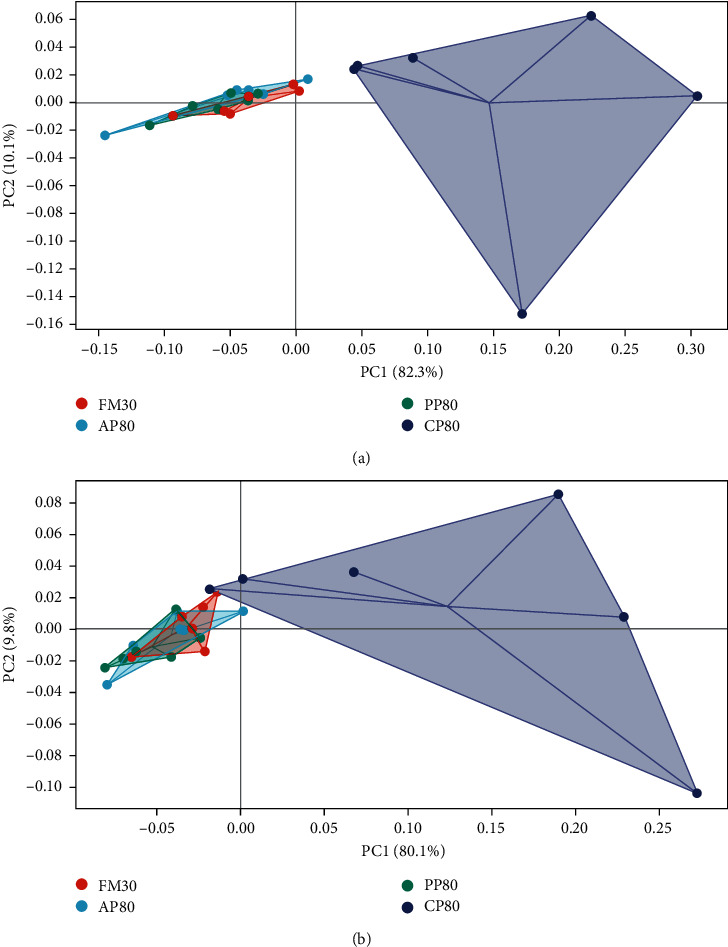
*β* Diversity of intestinal microbiota of juvenile golden pompano at the phylum (a) and genus (b) levels principal component analysis (PCA) against PC1 versus PC2 axes based on OTUs.

The results were shown in Table 8, no statistical difference was observed in the chao and coverage index among all the groups (*P* > 0.05). The shannon and simpson index of the CP80 group were significantly lower than those of the other groups (*P* < 0.05). In addition, principal component analysis (PCA) showed that the FM30, AP80, and PP80 groups had a partial overlap, while the CP80 group clustered away from the other three groups (Figure 1).

**Figure 2:**
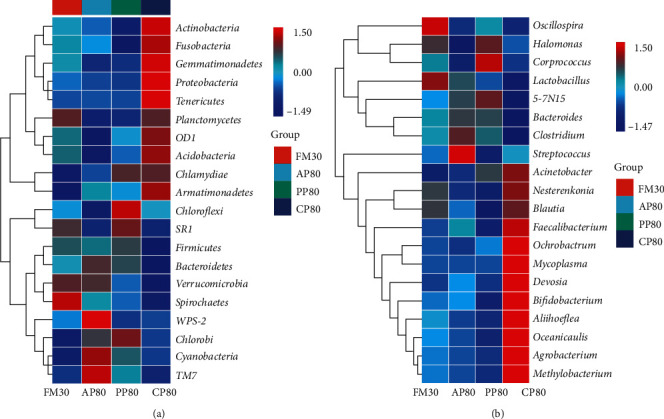
Heatmap of the abundance of golden pompano intestinal bacteria at phylum (a) and genus (b) levels in different experiment groups. Phylogenetic positions are projected by the OTUs, and the taxa of OTUs are listed on the right. Color intensity indicates the relative enrichment of OTUs.

**Figure 3:**
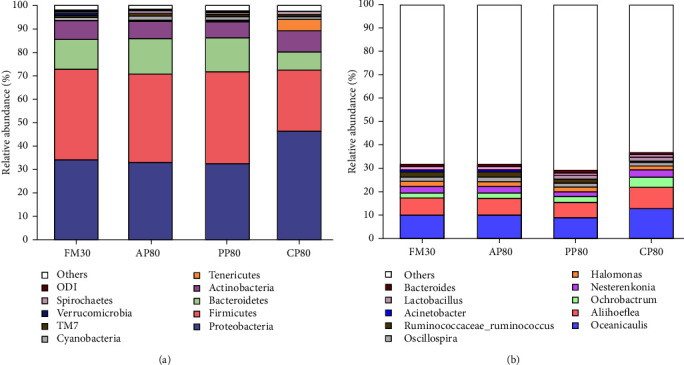
Intestinal bacterial composition at the phylum (a) and genus (b) levels in different experiment groups. Top 10 most abundant bacterial phyla and genera were shown in the figures, respectively. Other phyla and genera were all assigned as “Others”.

**Figure 4:**
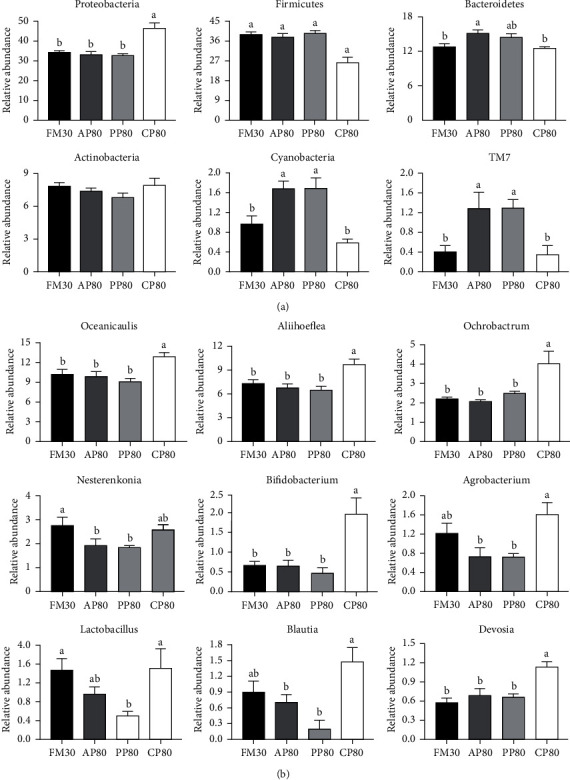
Phylum (a) and genus (b) level relative abundances of intestinal flora of juvenile golden pompano fed different diets for 10 weeks. Values presented as means ± SEM (*n* = 6). Bars assigned by different letters are significantly differed at *P* < 0.05.

The cluster heat maps reflected the distribution patterns of bacterial taxa in the intestine of golden pompano fed different diets (Figure 2). Across treatments, 20 phylum and 20 genus were observed in the intestinal microbiota. As it can be seen from Figures 3 and 4, no statistical difference (*P* > 0.05) was observed in the Actinobacteria (in phylum) and *Nesterenkonia* (in genus) among all the groups. At the phylum level, the relative abundances of proteobacteria, firmicutes, bacteroidetes, and actinobacteria were higher in all group, and the values of proteobacteria were significantly higher in group CP80, while firmicutes and bacteroidetes were significantly lower than those of the FM30, AP80, and PP80 groups (*P* < 0.05). The values of cyanobacteria and TM7 of the CP80 group showed no significant difference from those of the FM30 group (*P* > 0.05), but they were significantly lower than those of the AP80 and PP80 groups (*P* < 0.05). At the genus level, the relative abundances of *Oceanicaulis*, *Aliihoeflea*, *Ochrobactrum*, and *Nesterenkonia* were higher, and the relative abundances of *Oceanicaulis*, *Aliihoeflea*, *Ochrobactrum*, *Bifidobacterium*, and *Devosia* of the CP80 group were significantly higher than those of the FM30, AP80, and PP80 groups (*P* < 0.05). In addition, the *Agrobacterium* and *Blautia* of the CP80 group showed no significant difference from that of the FM30 group (*P* > 0.05), and they were significantly higher than those of the AP80 and PP80 groups (*P* < 0.05).

### 3.5. Intestinal Innate Immunity-Related Genes and Tight Junction Protein Genes Expression

**Figure 5:**
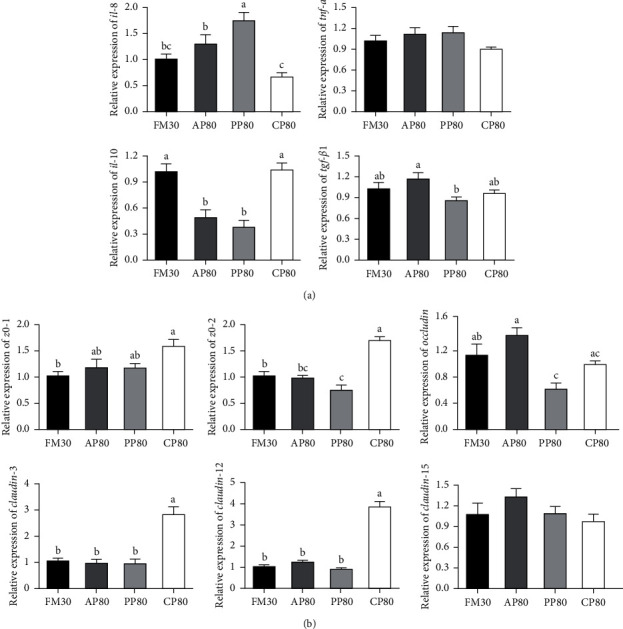
Relative mRNA expression of inflammation-related genes (a) and tight junction protein genes (b) in the intestine juvenile golden pompano fed different diets for 10 weeks. Values presented as means ± SEM (*n* = 6). Bars assigned by different letters are significantly differed at *P* < 0.05. il-8, interleukin-8; *tnf-α*, tumor necrosis factor-*α*; *il*-10, interleukin-10; *tgf-β*1, transforming growth factor-*β*1; *zo*-1, zonula occludens-1; *zo*-2, zonula occludens-2.

The genes expression associated with intestinal innate immunity and tight junction protein were shown in Figure 5. There were no significant differences in the *tnf-α* and *claudin*-15 expression among all groups (*P* > 0.05). The values of *il*-8 and *il*-10 of the FM30 group, *tgf-β*1 of the FM30, AP80, and PP80 groups, *zo*-1 of the AP80 and PP80 groups, as well as *occludin* of the FM30 and PP80 groups showed no significant difference with those of the CP80 group (*P* > 0.05). The values of *il*-8 of the AP80 and PP80 groups were significantly higher, while *il*-10 of the AP80 and PP80 groups, *zo*-2, *claudin*-3, and *claudin*-12 of FM30, AP80, and PP80 groups were significantly lower, compared with those of the CP80 group (*P* < 0.05).

### 3.6. Serum Growth Hormone Content

**Figure 6:**
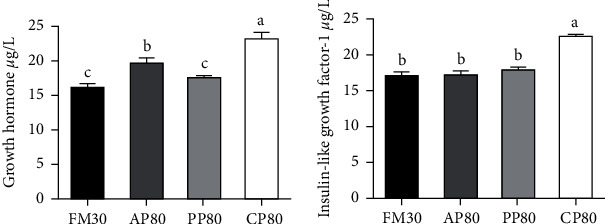
Serum growth hormone (*μ*g/L) and insulin-like growth factor-1 (*μ*g/L) content of juvenile golden pompano fed different diets for 10 weeks. Values presented as means ± SEM (*n* = 6). Bars assigned by different letters are significantly differed at *P* < 0.05.

The serum GH content was shown in Figure 6. The serum contents of GH and IGF-1 of the CP80 group were significantly higher than those of the other groups (*P* < 0.05).

### 3.7. Enzyme Activity and Gene Expression Related to Protein Metabolism

The enzyme activities related to protein metabolism in the serum and liver were shown in Table 9. There were no significant differences in serum T-AA content among all groups (*P* > 0.05). In serum, the AST, ALT, XOD, and SDH activities of the CP80 group showed no significant difference from those of the FM30 group (*P* > 0.05), the TP content was significantly higher than that of FM30 and AP80 groups, the XOD activity was significantly higher than that of AP80 group, SDH activity was significantly lower than that of the AP80 group, AST activity was significantly lower than that of the PP80 group (*P* < 0.05). In the liver, the ALT activity of CP80 group was not significantly different from that of the FM30 group (*P* > 0.05), and the TP content, as well as the activities of XOD and SDH were significantly higher than those of the FM30 group, the ALT activity was significantly higher than that of the AP80 and PP80 groups, the AST activity was significantly lower than that of the FM30 group (*P* < 0.05).

**Figure 7:**
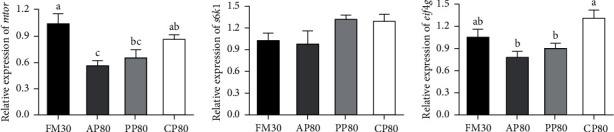
Relative expression of the mammalian target of rapamycin (*mtor*), ribosomal protein S6 kinase 1 (*s*6*k*1), and eukaryotic translation initiation factor 4G (*eif*4*g*) in the liver of juvenile golden pompano fed different diets for 10 weeks. Values presented as means ± SEM (*n* = 6). Bars assigned by different letters are significantly differed at *P* < 0.05.

The gene expression associated with protein metabolism was shown in Figure 7. There were no significant differences in the expression of *s*6*k*1 among all the groups (*P* > 0.05). The values of *mtor* and *eif*4*g* genes in liver of the CP80 group were not significantly different from those of FM30 group (*P* > 0.05), and *mtor* expression level was significantly higher than that of AP80 group, and *eif*4*g* expression level was significantly higher than that of the AP80 and PP80 groups (*P* < 0.05).

## 4. Discussion

After a 10 week feeding trial, 80% replacement of dietary FM (the diets containing only 6% FM) by the Cpro displayed no negative effects on the growth performance golden pompano, which are similar to our previous reports [25, 31]. However, the reason for the decreased growth performance caused by the substitution of 80% FM by animal protein sources may be the high ash content of the feed and the imbalance of amino acids [44, 45], which reduces the digestibility of the feed [46]. In fact, the AP80 diet had the highest ash content, and lower total EAAs compared with the other diets. The imbalance of amino acids in feed can significantly reduce the growth performance of aquatic animals, which has been verified in largemouth bass (*Micropterus salmoides*) [47], blunt snout bream (*Megalobrama amblycephala*) [21], and european sea bass (*Dicentrarchus labrax*) [48]. On the contrary, it has been shown that plant protein consisting of soybean meal, rapeseed meal, cottonseed meal, distillers dried grains with soluble, wheat gluten, soy protein concentrates, and corn gluten meal can completely replace FM in the diet without negatively affecting the growth of gibel carp (*Carassius auratus gibelio*) by adding lysine, methionine, and threonine to the diet to balance amino acids [49]. In the PP80 group, the results of growth performance may be attributed to the fact that there are high anti-nutritional factors in the feed, which reduces the digestibility of the feed and inhibits the growth of fish [50, 51]. In fact, the content of anti-nutritional factors in PP80 diets is higher than that in other diets (Table 3). Similar results were reported in some carnivorous fish, such as salmon trout (*Oncorhynchus mykiss*) [52], largemouth bass [53], and atlantic cod (*Gadus morhua*) [54], in which growth performance was significantly reduced with higher levels of anti-nutritional factors in the feed. As for the CP80 group, the reasonable explanation of good growth performance is probably due to the fact that Cpro has relatively low content of anti-nutritional factors (Table 3) and similar amino acid composition to FM (Table 2). Therefore, 80% of dietary FM was replaced by Cpro, which has better amino acid composition, lower content of anti-nutritional factors, and higher in vitro digestion rate, which can reduce the content of FM to 6% without reducing the growth performance of golden pompano, suggesting its good potential for application in production.

The intestine is a very important digestive and immune organ of fish, and a healthy intestine is beneficial for the growth and health of fish [55]. Intestinal enzyme activities were considered to be a reliable indicator of the intestinal digestive and absorptive capacities of fish, due to the fact that these enzymes determine the ability of fish to obtain and utilize nutrients in diets [56–59]. CHT is a proteolytic enzyme secreted by the pancreas, which can rapidly break down denatured proteins [60]. In this study, the activity of CHT in the foregut of the CP80 group was similar to that of the FM30 group, but lower than that of the AP80 and PP80 groups, possibly due to the fact that the meat and bone meal in AP80 diet contains bones that are difficult to digest [46], while the PP80 diet has a high content of anti-nutritional factors [51], so it is necessary to improve the digestive enzyme activity of fish to cope with indigestible substances. In fact, AP80 and PP80 diets had the lowest in vitro digestibility (Table 4). Pepsin (PEP) has the function of breaking down protein in food into small peptides [61]. The possible reason for the higher PEP activity in the foregut of the CP80 group is that the diet contains fermented soybean meal and hydrolyzed soybean meal (high content of small peptides), and the higher PEP activity is mainly for the digestion of more small peptides for growth function [62]. The possible reason for the higher activity of PEP in the foregut of the AP80 group is that the meat and bone meal in the AP80 feed contains bones that are difficult to digest, so the fish needs to secrete more PEP to cope with indigestible substances, similar results have also been reported in turbot (*Scophthalmus maximus*) [63]. In addition, the midgut PEP activity also reflected this phenomenon, the activity of the FM30 and CP80 groups was significantly lower than that of AP80 and PP80 groups, indicating that the two groups were more conducive to digestion and absorption, and the growth performance of fish in FM30 and CP80 groups was significantly higher than that in AP80 and PP80 groups, which also confirmed this view. The role of intestinal LPS is to hydrolyze fat to glycerol and fatty acids [64]. In this study, replacing 80% FM with plant protein increased intestinal LPS activity probably because plant protein is difficult to be decomposed and utilized [51], resulting in increased LPS activity to break down fat for energy, and the high-LPS activity in the midgut also supports this idea. The reason for the higher activity of LPS in the midgut of the CP80 group may be that it can provide sufficient energy with protein, so the fat utilization rate is low. Therefore, more fat in the foregut is not digested in the foregut, and more digestive enzymes are needed to be secreted by the midgut to digest fat. Similar results were also reported in turbot, that is, with the increase of plant protein content in the diet, the activity of intestinal digestive enzymes increased significantly [63]. These results suggested that CP80 diets can enhance intestinal digestive function, while AP80 and PP80 diets weakened it. In addition, the improvement of intestinal digestive function in the CP80 group possibly is due to that high-dietary leucine content, as it has been shown that leucine could promote the digestion and utilization efficiency of the digestive enzymes [65]. This result was also confirmed in largemouth bass [66], dusky kob (*Argyrosomus japonicus*) [67], and gilthead seabream (*Sparus aurata*) [13]. The intestinal microbiota consists of a dynamic organization of bacteria, viruses, archaea, and fungal species essential to maintain intestinal homeostasis and host health [68]. They are affected by the environment, feed composition, and the developmental stage of the host [39, 41]. In the study, CP80 intake resulted in a significant decrease in the diversity estimator (shannon and simpson), indicating a reduction in intestinal microbial diversity in fish in the CP80 group. In addition, approximately 90% of the total abundance of intestinal microbiota (in phylum) in this fish were composed of the four most abundant taxa: proteobacteria, firmicutes, bacteroidetes, and actinobacteria. Among these, proteobacteria, firmicutes, and bacteroidetes are dominant phyla that colonize the intestine of different fish species [69, 70]. According to the previous studies, the abundance variation of bacteroidetes, firmicutes, and proteobacteria in the intestine of aquatic animals could be affected by the culture environment and the composition of nutrients [71, 72]. The relative abundances of these three bacterial classes in the study indicated how well golden pompano was adapted to different FM replacement sources in the diets. The current study has shown that the highest values of bacteroidetes and firmicutes were found in the groups AP80 and PP80, respectively. Previous studies have shown that bacteroidetes can promote carbohydrate fermentation, thus improving host intestinal digestive function [73]. Firmicutes can produce fatty acids to enhance the innate immune system, thereby improving the health of intestinal mucosa [74, 75]. Furthermore, relatively high values of actinobacteria and proteobacteria were found in the CP80 group, suggesting that the intake of CP80 diets could enhance the immune function of fish. This may be due to the fact that actinobacteria can enhance the ability of host defense against pathogenic invasions by colonizing the intestine to form a defensive barrier [38]. In general, *Bifidobacterium*, *Blantia*, and *Devosia* can improve immunity and growth performance of the living organisms [76–78]. In this study, fed CP80 fish have higher relatively abundance of the beneficial bacteria *Bifidobacterium*, *Lactobacillus*, *Devosia*, and *Blantia*. This further supported that CP80 diets can improve the intestinal flora of golden pompano. Interestingly, in this study, feeding diet CP80 increased the relative abundance of *Oceanicaulis*, *Aliihoeflea*, and *Ochrobactrum* in the gut of golden pompano, but its function in aquatic animals is less reported and unclear. When pathogens invade fish, nonspecific immunity first acts as a defense to maintain body health [79, 80]. In the study, the expression of *il*-8 and *tnf-α* expression of AP80 and PP80 groups was higher than that of the FM30 group, but the opposite was true for the expression of *il*-10. The results suggested that the intake of both AP80 and PP80 diets can induce an inflammatory response in the intestine. This was supported by the fact that pro-inflammatory cytokines (such as *il*-8 and *tnf-α*) are the sensitive mediators of the host immune response [80, 81]. For example, infection of pathogenic bacteria usually stimulates the expression of pro-inflammatory cytokines [82]. In this study, diet PP80 contains plant protein, and a high concentration of plant protein instead of FM has been confirmed to cause an intestinal inflammatory reaction in the aquatic animals [80]. In addition, the highest value of *il*-10, but the lowest values of *il*-8 and *tnf-α* were found in the CP80 group, further confirming that the intake of CP80 diets can enhance the immune function of fish. Additionally, the physical barrier function of the fish intestine also depends on the integrity of the intercellular connection [83]. In the study, the expression of *zo*-2, *claudin*-3, and *claudin*-12 in the CP80 group was higher than in the other groups. This indicates that feeding CP80 can significantly improve fish intestinal health, which is related to low-anti-nutritional factors and amino acid balance in the feed [45, 49, 84]. Therefore, 80% of dietary FM replaced by Cpro can improve the activity of intestinal digestive enzymes, immunity responses, and the structure of the intestinal microbiota of golden pompano.

The three interrelated processes of protein synthesis, degradation, and growth are called protein metabolism [85, 86]. Growth is achieved mainly through protein synthesis and retention, in which enzymes related to protein metabolism play an important role [87]. For example, the content of serum total protein (TP) can reflect the ability of aquatic animals to absorb and metabolize protein, which is positively correlated with growth performance [88]. ALT and AST are the most widely distributed and most active transaminases in the body [89], and when the liver function of fish is impaired or damaged, ALT and AST will be released into the blood, resulting in increased serum transaminase activity and affecting protein metabolism [90, 91]. In this study, the highest TP content in fish serum was found in PP80 and CP80 groups, and the growth performance of fish in the CP80 group was significantly higher than that in the PP80 group (Table 6), owing to the high content of anti-nutritional factors in diet PP80 (Table 3), which cannot be efficiently digested and utilized, and this result was also reflected in the digestive enzyme activity of the intestine (Table 7). AST activity in serum in the PP80 group was significantly increased but the opposite trend was observed in the liver, indicating that a high content of plant protein in the diet would cause damage to liver cells, and transaminases were released, resulting in decreased protein metabolism [90]. The main reasons are high content of amino acid imbalance (Table 2) and anti-nutritional factors (Table 3) in the diet, which have also been verified in largemouth bass [66] and japanese seabass (*Lateolabrax japonicus*) [89]. However, serum AST activity in the CP80 group was significantly lower than that in the PP80 group, indicating that 80% of dietary FM replaced by Cpro can improve protein metabolism in fish, which may be related to the balanced amino acid compositions and low content of anti-nutritional factors in the diet (Tables 2 and 3), which can improve the digestibility of golden pompano (Table 4). Succinic dehydrogenase (SDH), a key mitochondrial inner membrane enzyme involved in the energy-producing citric acid cycle in living cells [92], has been used as a biochemical marker in inflammation. XOD is one of the enzymes involved in the generation of reactive oxygen species (ROS) and causes damages [93]. In the present study, feeding a diet that 80% of the dietary FM replaced by animal protein significantly decreased the activity of XOD in serum and increased the activity of SDH in the liver, which may be related to low-anti-nutritional factors in the diet [50]. Protein synthesis is an important part of the growth reaction, which is mainly regulated by targets of rapamycin (*mtor*) signaling pathway [75, 94]. The TOR signaling pathway promotes protein synthesis in fish and mammals through *eif*4*g* and *s*6*k*1 [95]. However, the TOR pathway is regulated by growth factors [75, 96]. In this study, the serum GH and IGF-1 content in the CP80 group was significantly higher than that in the other groups (Figure 6), and the expression levels of the genes *mtor* and *eif*4*g* in liver tissue were not significantly different from those of the control group, but they were significantly higher than those of the AP80 and PP80 group (Figure 7). Similar results have been reported in several carnivorous fish, such as turbot [63], japanese seabass [97], and largemouth bass [98], which can promote growth by activating the mTOR pathway. In addition, dietary amino acids such as threonine, isoleucine, and leucine can also increase serum growth factor content and TOR gene expression [99, 100]. In fact, the leucine possessed highest content in the CP80 diet (Table 2). It has also been reported in the study of golden pompano that with the increase of the proportion of FM replaced by nongrain Cpro (NGCP, bovine bone meal, dephenolized cottonseed protein, and blood cell meal), the contents of lysine and methionine in the feed decreased, and the lack of this nutrient may inhibit the mTOR signaling pathway, resulting in the obstruction of protein synthesis, which in turn affected the growth of fish [101]. In addition, many studies have found that dietary anti-nutritional factors also reduce serum growth factor content and inhibit *tor* gene expression [102]. Here, the study also confirmed this view, due to we found the expressions of the *mtor* and *eif*4*g* in the PP80 group (it has containing the highest anti-nutritional factors contents) were significantly lower than those of the control group. These results indicated that the replacement of FM with a high proportion of Cpro had no effects on the expression of genes related to TOR signaling pathway, while the replacement of FM with a high proportion of animal protein or plant protein showed negative effects. Therefore, 80% of dietary FM replaced by Cpro can improve the protein metabolism of golden pompano.

## 5. Conclusion

In summary, the present study showed that after 80% of dietary, FM was replaced by Cpro, which lead to the dietary FM content of as low as 6% (CP80), the dietary EAAs, anti-nutritional factors contents, and in vitro digestion rate were similar to those of the control diet containing 30% FM (FM30). Golden pompano fed with diet CP80 displayed better performance through the enhancement of the digestive enzyme activity, immunity response, protein metabolism, and the abundance of the beneficial bacteria of intestinal microbiota, these factors may be the important reasons that FM be replaced by Cpro with a high proportion in the diet of golden pompano.

## Figures and Tables

**Table 1:** Composition and nutrient levels of experimental diets (% dry weight).

Ingredients	Diets			
	FM30^a^	AP80^b^	PP80^c^	CP80^d^
Fishmeal	30.00	6.00	6.00	6.00
Basic protein^e^	25.00			
Animal protein^f^		59.00		
Plant protein^g^			60.00	
Compound protein^h^				57.00
Compound oil^i^	7.60	4.00	9.50	8.00
Gluten flour	17.00	17.00	17.00	17.00
Premix compound^j^	2.00	2.00	2.00	2.00
*L*-lysine		0.35	0.43	0.48
Shotcrete corn husk	18.40	11.65	5.07	9.52
Total	100.00	100.00	100.00	100.00
Proximate composition (%)				
Dry matter	87.21	87.62	88.26	92.97
Crude protein	45.12	45.28	45.13	45.11
Crude lipid	12.20	12.21	12.37	12.23
Crude fiber	3.02	2.62	3.18	3.05
Ash	9.80	16.33	6.59	8.71

*Note*. The vitamin premix and mineral premixes were purchased from Guangdong Yuehai Feeds Group Co. Ltd., China, while the compound oil was provided by Guangzhou UBT Feed Technology Co. Ltd., China, and other feed ingredients were purchased from Taishan Xiangxing Feed Co. Ltd., China. ^a^FM30: control, contained 30% fishmeal. ^b^AP80: animal protein completely replaces 80% of fishmeal and 25% of base protein. ^c^PP80: plant protein completely replaces 80% of fishmeal and 25% of base protein. ^d^CP80: compound protein completely replaces 80% of fishmeal and 25% of base protein. ^e^Basic protein was composed of poultry meal:soy protein concentrate:corn gluten meal = 7 : 12 : 6, with a crude protein content of 51.62% and a crude lipid content of 3.86%. ^f^Animal protein was composed of poultry meal: meat and bone meal = 30 : 29, with a crude protein content of 59.24% and a crude lipid content of 12.32%. ^g^Plant protein was composed of soybean protein concentrate : corn gluten meal : fermented soybean meal : peanut meal = 12 : 12 : 28 : 8, with a crude protein content of 60.46% and a crude lipid content of 2.92%. ^h^The specific composition and proportion of compound protein was not shown due to the protection of patent granted by Republic of South Africa (patent No. 2023/00178), which had a content of 54.11% crude protein and 6.34% crude lipid, respectively. ^i^Consists of fish, soybean, rapeseed, perilla oils and phospholipid in different proportion, together with small amount of emulsifier and antioxidant [35]. ^j^Consists of ethoxyquinoline, choline chloride, monocalcium phosphate, betaine, vitamin mixture and mineral premixes. Vitamin supplied per kilogram diet: VA, 11,00,000 IU; VD_3_, 3,20,000 IU; VE, 80 mg; VB_12_, 8 mg; VC, 120 mg; VK_3_, 1,000 mg; VB_1_, 1,500 mg; VB_2_, 2,800 mg; calcium pantothenate, 2,000 mg; nicotinamide, 7,800 mg; folic acid, 400 mg; inositol, 12,800 mg; VB_6_, 1,000 mg. Mineral premixes supplied per kilogram diet: sodium fluoride, 2 mg; potassium iodide, 0.8 mg; cobalt chloride (1%), 50 mg; copper sulfate, 10 mg; zinc sulfate, 50 mg; manganese sulfate, 60 mg; magnesium sulfate, 1,200 mg; sodium chloride, 100 mg; zeolite powder: 15.45 g.

**Table 2:** Amino acids composition of the experimental diets (g/100 g dry weight).

Amino acids (AA)	Diets			
	FM30	AP80	PP80	CP80
Essential amino acids (EAAs)				
Threonine	1.82	1.67	1.72	1.67
Valine	2.10	1.98	1.96	1.98
Methionine	0.89	0.76	0.64	0.75
Isoleucine	1.77	1.47	1.75	1.66
Leucine	3.69	3.08	4.07	4.14
Phenylalanine	1.92	1.63	2.22	2.09
Lysine	2.65	2.70	2.24	2.31
Histidine	1.22	0.90	1.04	1.00
Arginine	2.56	3.01	2.81	2.67
Total EAAs	18.60	17.21	18.47	18.27
None essential amino acids (NEAAs)				
Aspartic acid	3.75	3.35	4.17	3.69
Serine	1.94	1.88	2.21	2.12
Glutamic acid	7.41	6.54	8.89	8.40
Glycine	2.40	4.42	1.93	2.52
Alanine	2.80	3.16	2.54	2.91
Cystine	0.41	0.33	0.47	0.49
Tyrosine	1.41	1.20	1.64	1.55
Proline	2.71	3.05	2.96	3.22
Total NEAAs	22.82	23.93	24.82	24.90

*Note*. Data are mean of three duplicates determination. Tryptophan was not determined in the present study.

**Table 3:** Anti-nutritional factors content of the experimental diets (dry weight).

Anti-nutritional factors	Diets			
	FM30	AP80	PP80	CP80
*β*-Conglycinin (*μ*g/g)	320.93	320.78	515.72	446.53
Soybean antigen (*μ*g/g)	1623.87	1373.66	2074.81	1468.70
Soy glycinin (*μ*g/g)	1318.06	1364.69	2030.29	1490.55
Phytagglutinins (ng/g)	6.91	5.90	8.87	7.05
Plant trypsin inhibitor (ng/g)	0.32	0.32	0.43	0.35

*Note*. Data are mean of six duplicates determination.

**Table 4:** In vitro digestion rate of the experimental diets (dry weight).

In vitro digestion rate	Diets			
	FM30	AP80	PP80	CP80
Dry matter digestibility^1^	79.15	78.25	77.47	81.47
Protein digestibility^2^	78.84	73.00	68.16	77.79
Lipid digestibility^3^	34.03	30.28	25.13	32.03

*Note*. Data are mean of three duplicates determination. ^1^Dry matter digestibility (%) = 100 × (feeds sample weight (g)−indigestible residue weight (g))/feeds sample weight (g). ^2^Protein digestibility (%) = 100 × (total protein in sample (%)−digestible protein (%))/total protein in sample (%). ^3^Lipid digestibility (%) = 100 × (total lipid in sample (%)−digestible lipid (%))/total lipid in sample (%).

**Table 5:** Nucleotide sequences of the primers used to assay gene expressions using real-time PCR.

Gene name	Forward primer (5′-3′)	Reverse primer (3′-5′)	Reference
*mtor*	GATCAGGAGAGAGGCCATCC	AGCCGGGTAAAACTCATCCA	MW846078.1
*s*6*k*1	GAAGCCCAAGAACACCTGTG	GCTTGTGTCCATTTGCTCCA	XM_030438737.1
*eif*4*g*	AAAGTCCGAGAATGCCTGGA	CGTCGATGGCCTTCTCAAAG	XM_044349829.1
*il*-8	TGCATCACCACGGTGAAAAA	GCATCAGGGTCCAGACAAATC	[42]
*il*-10	CTCCAGACAGAAGACTCCAGCA	GGAATCCCTCCACAAAACGAC	[42]
*tnf-α*	CATCAATGCTGCTAGGCTGG	TAGAAGGTGCATCGTGGTGT	[42]
*tgf-β*1	GAGATACGGAAAAGAGTGGGG	TGACAAAGCGGGAAGCAAG	[42]
*zo*-1	AATGATGGTTGGTTTGGGGC	CTCTGTGTCTGTGTCCTCGT	XM_020606686.1
*zo*-2	GGGGAAGGTCAAGGCTTTTG	GGCGGTGTGATATCCTGGTA	Genome sequences
*occludin*	CTCCCCTCAGAGCCAGTATG	CATGTCCCACACCAAGGTTG	XM_030403522.1
*claudin*-3	CCTCTCCCAAGACCTCCAAG	GATTATGGTGTTGGCCGACC	XM_030440595.1
*claudin*-12	GGCTGGGATGTGTAAGACCT	TAGTCCTCCAGAGCAGCCTA	XM_020093230.1
*claudin*-15	CAAAGGCAGGAGGGGAAAAC	CAGCCGATGTAAAGCCCTTC	XM_003449343.5
*β-actin*	TACGAGCTGCCTGACGGACA	GGCTGTGATCTCCTTCTGC	[42]

*Note*. *mtor*, mammalian target of rapamycin; *s*6*k*1, ribosomal protein S6 kinase 1; *eif*4*g*, eukaryotic translation initiation factor 4 G; *il*-8, interleukin-8; *il*-10, interleukin-10; *tnf-α*, tumor necrosis factor-*α*; *tgf-β*1, transforming growth factor-*β*1; *zo*-1, zonula occludens-1; *zo*-2, zonula occludens-2. The *zo*-2 primers were designed according to the genome sequences of golden pompano (10.6084/m9.figshare.7570727.v3).

**Table 6:** Growth performance, feed utilization, and somatic parameter of juvenile golden pompano fed different diets for 10 weeks.

Groups	FM30	AP80	PP80	CP80
Growth performance				
IBW (g)	10.50 ± 0.10	10.11 ± 0.06	10.44 ± 0.22	10.22 ± 0.22
FBW (g)	105.63 ± 1.90^a^	90.06 ± 0.04^b^	88.38 ± 0.97^b^	102.39 ± 0.52^a^
WG^1^ (%)	906.37 ± 25.56^a^	790.77 ± 4.61^b^	755.88 ± 18.26^b^	902.74 ± 25.33^a^
SR^2^ (%)	100.00 ± 0.00	100.00 ± 0.00	100.00 ± 0.00	98.89 ± 1.11
Feed utilization				
FCR^3^	1.14 ± 0.02^c^	1.36 ± 0.00^ab^	1.47 ± 0.07^a^	1.25 ± 0.01^bc^
Somatic parameter				
VSI^4^ (%)	5.86 ± 0.09^b^	6.71 ± 0.11^ab^	6.94 ± 0.25^a^	6.53 ± 0.31^ab^
HSI^5^ (%)	1.05 ± 0.12	1.32 ± 0.07	1.12 ± 0.06	1.13 ± 0.10
CF^6^ (g/cm^3^)	3.14 ± 0.11	3.21 ± 0.11	3.37 ± 0.08	3.43 ± 0.12

*Note*: Growth performance and feed utilization results are presented as the means ± SEM of three replications. Somatic parameter results are presented as the means ± SEM of six replications. Values in the same line with different superscripts are significantly different (*P* < 0.05). IBW (initial mean body weight, g fish^−1^); FBW (final mean body weight, g fish^−1^). ^1^ Weight gain (WG, %) = 100 × (wet weight (g)−initial weight (g))/initial weight (g). ^2^ Survival rate (SR, %) = 100 × (final number of fish)/(initial number of fish). ^3^ Feed conversion ratio (FCR) = feed consumed (g)/(wet weight (g)−initial weight (g)). ^4^ Viscerosomatic index (VSI, %) = 100 × viscera wet weight (g)/final body weight (g). ^5^ Hepatosomatic index (HSI, %) = 100 × liver wet weight (g)/final body weight (g). ^6^ Condition factor (CF, g/cm^3^) = 100 × final body weight (g)/body length (cm) ^3^.

**Table 7:** Intestinal enzyme activity of juvenile golden pompano fed different diets for 10 weeks.

Groups	FM30	AP80	PP80	CP80
Foregut				
PEP (U mgprot^−1^)	2.54 ± 0.21^b^	3.64 ± 0.27^a^	2.23 ± 0.20^b^	3.67 ± 0.23^a^
CHT (U mgprot^−1^)	3.18 ± 0.64^b^	4.98 ± 0.53^a^	5.66 ± 0.58^a^	2.36 ± 0.41^b^
LPS (U mgprot^−1^)	1.43 ± 0.27^ab^	0.95 ± 0.16^b^	1.62 ± 0.07^a^	1.45 ± 0.23^ab^
AMS (U mgprot^−1^)	0.32 ± 0.02^bc^	0.36 ± 0.01^a^	0.35 ± 0.01^a^	0.29 ± 0.01^c^
Midgut				
PEP (U mgprot^−1^)	0.60 ± 0.12^c^	1.31 ± 0.13^ab^	1.51 ± 0.24^a^	0.91 ± 0.15^bc^
CHT (U mgprot^−1^)	1.66 ± 0.31^b^	1.50 ± 0.24^b^	3.17 ± 0.39^a^	1.48 ± 0.34^b^
LPS (U mgprot^−1^)	2.47 ± 0.53^c^	3.38 ± 0.61^bc^	6.00 ± 0.78^a^	5.10 ± 0.68^ab^
AMS (U mgprot^−1^)	0.39 ± 0.02^c^	0.46 ± 0.02^b^	0.67 ± 0.03^a^	0.64 ± 0.02^a^
Hindgut				
PEP (U mgprot^−1^)	2.41 ± 0.45^a^	0.46 ± 0.07^b^	2.43 ± 0.29^a^	1.02 ± 0.26^b^
CHT (U mgprot^−1^)	3.57 ± 0.37^a^	1.60 ± 0.40^b^	3.26 ± 0.32^a^	2.79 ± 0.27^a^
LPS (U mgprot^−1^)	5.18 ± 0.75^a^	2.61 ± 0.41^b^	3.11 ± 0.43^b^	2.68 ± 0.27^b^
AMS (U mgprot^−1^)	0.46 ± 0.01^b^	0.39 ± 0.01^c^	0.44 ± 0.00^b^	0.58 ± 0.01^a^

*Note*. Values are means ± SEM of six replications. Values in the same line with different superscripts are significantly different (*P* < 0.05). PEP, pepsin; CHT, chymotrypsin, U mgprot^−1^; LPS, lipase; AMS, *α*-amylase.

**Table 8:** Changes of alpha diversity in intestinal microbiota of juvenile golden pompano fed with different diets.

Groups	FM30	AP80	PP80	CP80
Coverage	1.00 ± 0.00	1.00 ± 0.00	1.00 ± 0.00	1.00 ± 0.00
Chao1	747.68 ± 31.69	712.63 ± 18.39	668.90 ± 18.94	706.77 ± 28.19
Simpson	0.98 ± 0.00^a^	0.98 ± 0.00^a^	0.98 ± 0.00^a^	0.96 ± 0.00^b^
Shannon	7.71 ± 0.10^a^	7.63 ± 0.05^a^	7.65 ± 0.07^a^	6.78 ± 0.20^b^

*Note*. Values are means ± SEM of six replications. Values in the same line with different superscripts are significantly different (*P* < 0.05).

**Table 9:** Serum and liver enzymes related to protein metabolism of juvenile golden pompano fed different diets for 10 weeks.

Groups	FM30	AP80	PP80	CP80
Serum				
TP (g/L)	36.72 ± 0.77^b^	39.01 ± 1.21^b^	42.78 ± 1.61^a^	42.59 ± 0.90^a^
T-AA (*μ*mol/L)	36.27 ± 1.87	39.32 ± 2.79	36.90 ± 1.66	38.55 ± 1.44
AST (U/L)	1.85 ± 0.37^b^	1.83 ± 0.32^b^	8.60 ± 0.32^a^	1.75 ± 0.30^b^
ALT (U/L)	8.91 ± 0.02^ab^	8.98 ± 0.01^a^	8.85 ± 0.04^b^	8.93 ± 0.02^a^
XOD (U/L)	7.14 ± 0.50^a^	5.74 ± 0.28^b^	6.74 ± 0.37^ab^	6.98 ± 0.27^a^
SDH (U/mL)	11.45 ± 0.37^ab^	12.91 ± 0.87^a^	11.83 ± 0.76^ab^	10.08 ± 0.47^b^
Liver				
TP (g/L)	59.76 ± 2.80^b^	69.93 ± 1.88^a^	68.73 ± 1.26^a^	67.42 ± 2.38^a^
T-AA (*μ*mol/gprot)	7.19 ± 0.49^a^	5.87 ± 0.62^a^	3.33 ± 0.40^b^	4.33 ± 0.43^b^
AST (U/gprot)	16.09 ± 0.83^a^	12.52 ± 0.41^b^	15.25 ± 1.37^a^	11.68 ± 0.80^b^
ALT (U/gprot)	1.19 ± 0.01^ab^	1.17 ± 0.01^b^	1.17 ± 0.01^b^	1.21 ± 0.01^a^
XOD (U/gprot)	6.63 ± 0.40^b^	8.33 ± 0.59^b^	10.22 ± 0.69^a^	10.29 ± 0.64^a^
SDH (U/mgprot)	4.00 ± 0.34^c^	8.40 ± 0.63^a^	5.21 ± 0.53^bc^	5.97 ± 0.33^b^

*Note*. Values are means ± SEM of six replications. Values in the same line with different superscripts are significantly different (*P* < 0.05). TP, total protein; T-AA, total amino acids; AST, aspartate aminotransferase; ALT, alanine transaminase; XOD, xanthine oxidase; SDH, succinic dehydrogenase.

## Data Availability

All the data used to support the findings of this study are included in this paper.
